# Tryptophan in the diet ameliorates motor deficits in a rotenone‐induced rat Parkinson's disease model via activating the aromatic hydrocarbon receptor pathway

**DOI:** 10.1002/brb3.2226

**Published:** 2021-06-09

**Authors:** Yilin Wang, Shuangxi Chen, Jian Tan, Yijiang Gao, Hongye Yan, Yao Liu, Shanqing Yi, Zijian Xiao, Heng Wu

**Affiliations:** ^1^ Department of Neurology The First Affiliated Hospital University of South China Hengyang PR China; ^2^ Affiliated Nanhua Hospital University of South China Hengyang PR China

**Keywords:** aromatic hydrocarbon receptor pathway, dietary tryptophan, Parkinson's disease

## Abstract

**Background and purpose:**

Parkinson's disease (PD), a common neurodegenerative disorder with motor and nonmotor symptoms, does not have effective treatments. Dietary tryptophan (Trp) supplementation has potential benefits for the treatment of multiple disorders. However, whether additional Trp in the diet could be beneficial for PD remains to beinvestigated. In the present study, the neuroprotective role of dietary Trp on a rotenone‐induced rat model of PD was determined.

**Methods:**

The rotenone was injected to build the PD model, and then the rats were treated with Trp in the diet. And then, an open field test, western blot analysis, and enzyme linked immunosorbent assay (ELISA) were performed.

**Results:**

We observed that dietary Trp significantly ameliorated impaired motor function, upregulated tyrosine hydroxylase expression, inhibited the nuclear transport of Nuclear factor‐kappa B (NF‐κB) in substantia nigra (SN), and downregulated the protein levels of IL‐1β, IL‐6, and TNF‐α in serum in rotenone‐treated rats. However, these patterns were reversed in response to treatment with ampicillin, an agent that can clean intestinal Trp metabolism flora. Moreover, after using CH223191, an inhibitor of the aromatic hydrocarbon receptor (AhR) pathway, dietary Trp could not exert neuroprotective roles in the rotenone‐induced rat model of PD.

**Conclusion:**

These results suggest that Trp in the diet can protect against rotenone‐induced neurotoxicity to ameliorate motor deficits, which may be mediated through activating AhR pathway.

## Graphical abstract

In the present study, the neuroprotective role of dietary Trp on a rotenone‐induced rat model of PD was determined. We revealed that Trp in the diet can protect against rotenone‐induced neurotoxicity to ameliorate motor deficits, which may be mediated through activating AhR pathway.

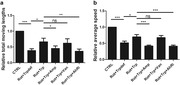

## 1 INTRODUCTION

Parkinson's disease (PD) is the second most common neurodegenerative disease, and is increasingly receiving attention from multiple neurologists (Pfeiffer, 2016). In this aging disorder, the degeneration of dopaminergic neurons was observed in substantia nigra pars compacta (SNpc), and resultant depletion of dopamine was detected in the striatum, accompanied by both motor and nonmotor symptoms (Dauer & Przedborski, 2003). A pro‐inflammatory profile was detected in the microbiota of PD patients (Bedarf et al., 2017; Petrov et al., 2017) owing to the increased intestinal permeability to lipopolysaccharide (LPS, an endtoxin) (Forsyth et al., 2011). To date, drug treatments for PD provided only be symptomatic, without preventing the progressive loss of dopaminergic neurons in patients with PD (Athauda & Foltynie, 2015). In consequence, research on an agent that may target for the neuroinflammation to treat PD is necessary.

Attempts to target individual molecules that may decrease the pathological impact of PD have been made to counteract dopaminergic neuron death by the introduction of neuroprotective molecules. One of these targets is tryptophan (Trp), an essential amino acid mainly obtained from dietary intake in humans and only in part from endogenous protein degradation (Le Floc'h et al., 2011). Trp is involved in the development of brain areas associated with behavioral functions (Zhang et al., 2006). Moreover, rats with decreased intake of tryptophan had higher running performance (Yamamoto & Newsholme, 2003). Trp metabolites acting on the metabolic crossroad interconnecting different organs exert various beneficial effects (Le Floc'h et al., 2011; Roager & Licht, 2018) in a multiple of disorders, including central nervous system disorders, autoimmune diseases, and cancer (Zhou et al., 2019). Under inflammatory conditions, most Trp is diverted to produce Kyn and its metabolites kynurenine acid (KYNA) (Keszthelyi et al., 2012) with neuroprotective roles (Lim et al., 2017). Thus, dietary Trp may result in the generation of drugs for the treatment of multiple diseases (Platten et al., 2019), including PD.

However, since multiple underlying mechanisms are involved in PD, we explored other targets associated with dietary Trp that could ameliorate the pathology of PD. Aryl hydrocarbon receptor (AhR), binding to a variety of ligands, including xenobiotic ligands such as physiological compounds derived from the digestion of dietary components by commensal microbiota (Zhou, 2016) such as dietary Trp supplementation (Liang et al., 2018), was selected. The AhR, broadly expressed in immune cells (Lee et al., 2017), plays a pleiotropic role in maintaining both the innate and adaptive immune systems in multiple organs and has been shown to be a transcriptional regulator for the development and function of several immune cells (Stockinger et al., 2014). Also, AhR can inhibit the production of IL‐6 in response to the treatment of LPS via interacting with NF‐κB and STAT1 (Kimura et al., 2009). AhR signaling regulates multiple cellular processes, including immunomodulation, cell development, differentiation, proliferation, survival, and apoptosis (Stockinger et al., 2014). In cerebellar granule neuron precursors, disrupted expression of the AhR impairs neurogenesis via inhibiting precursor proliferation and increasing differentiation (Dever et al., 2016). In addition, constitutive activity of AhR is essential for spontaneous movement in mice (Williams et al., 2014).

Given the key neuroprotective roles of Trp and AhR signaling under inflammatory conditions, we were interested in their relationship with PD, hypothesizing that Trp in the diet may play a neuroprotective role in PD by fostering neuroinflammation inhibition‐associated signaling via AhR. To achieve this goal, rotenone‐challenged rats were used as an in vivo model of PD, and the effect of dietary Trp on motor function, dopaminergic neuronal survival and inflammation, and the underlying mechanisms associated with the AhR signaling pathway inhibited by CH223191 were investigated. Here, we revealed a neuroprotective role of dietary Trp against rotenone‐induced neurotoxicity via activating the AhR pathway.

## 2 MATERIALS AND METHODS

### 2.1 Animals

Male Sprague–Dawley (SD) rats (200–220 g) of 6 weeks of age were purchased from Hunan SJA Laboratory Animal Co., Ltd. and maintained (*n* = 4 rats/cage) in an air‐conditioned room (22 ± 1˚C) with a 12‐h light/12‐h dark cycle and water and food ad libitum. All experimental protocols performed on animals were approved by the Laboratory Animal Ethics Committee of the First Affiliated Hospital of University of South China.

### 2.2 Groups and treatments

Rats were randomly divided into six groups (*n* = 6 rats/group): Normal saline‐treated group (Control, CTRL), rotenone + Trp‐deficient‐treated group (Rot+Trpdef), rotenone + Trp‐treated group (Rot+Trp), rotenone + Trp + ampicillin‐treated group (Rot+Trp+Amp), rotenone + Trp + vancomycin‐treated group (Rot+Trp+Van), and rotenone + Trp + CH223191‐treated group (Rot+Trp+AhRi).

The rats in the CTRL group were treated daily with 0.1 ml saline for 4 weeks by subcutaneous injection through the neck and fed with normal diet. The rats in the other groups were subcutaneously injected with rotenone at a concentration of 2 mg/kg in the neck. The rats in Rot+Trpdef group were fed with Trp‐deficient forage. The left four groups were fed with Trp‐rich forage. Treatments with 300 mg/kg ampicillin or 150 mg/kg vancomycin by oral administration were performed at week four after the injection of rotenone. The rats in Rot+Trp+AhRi group were treated with 10 mg/kg CH223191 (cat no. A8609; APeXBIO Technology LLC) by oral administration at the week 1 after rotenone injection.

### 2.3 Open field test

An automated open field apparatus (Accuscan Instruments Inc.) was used to measure the locomotor activity of rats, as described previously (Gordon et al., 2016). The open field analysis box consists of a wooden open field box of 100 × 100 × 40 cm (length x width x height). The bottom of the box is divided into 25 squares (20 × 20 cm) on average. The grid is called the peripheral area, and the remaining area comprises the central area. A camera is placed in the middle of the open field box and connected to a computer. A rat is placed in the central compartment. The movement of the rat within 5 min is observed, and the total distance of movement is calculated. Two independent investigators, who were blinded to the experimental groups, recorded and quantified the total distance traveled. No animals died during the survival time, after which the rats were sacrificed.

### 2.4 Tissue preparation

The tissues were prepared according to previous studies (Chen et al., 2019; Li et al., 2017; Xu et al., 2017). For western blot analysis, rats were sacrificed by decapitation after 10% chloral hydrate anesthesia at a concentration of 350 mg/kg. Briefly, in the SNc area, three different levels (−4.8, −5.04, and −5.28 mm of bregma) were selected (Javed et al., 2020). SN tissues (*n* = 6/group) were collected and treated as follows: for extracting total proteins, SN tissue samples were dissolved in 100 μl RIPA buffer with 1% phenylmethylsulphonyl fluoride (PMSF) and homogenized with a micro‐tissue grinder (cat. no. 749540‐0000; Kimble Chase Life Science and Research Products, LLC). After centrifugation at 14,000 × *g* at 4˚C for 15 min, the supernatant containing total proteins was collected and stored at −80˚C. For extracting cytosolic proteins, samples were mechanically lysed in 1 ml ice‐cold buffer A [10 mM HEPES (pH 7.9), 2 mM MgCl_2_, 10 m MKCl, 0.1 mM EDTA, 1 mM dithiothreitol and 0.5 mM PMSF], followed by addition of 10% NonidetP‐40 solution. After being vortexed for 30 s and centrifuged for 10 min at 5000 × *g* at 4˚C, the cytosolic fraction extracts were collected and stored at −80˚C. The crude nuclear pellets were suspended in 200 μl ice‐cold buffer B [20 mM HEPES (pH 7.9), 1.5 mM MgCl_2_, 420 mM NaCl, 0.1 mM EDTA, 1 mM dithiothreitol, 0.5 mM PMSF, and 25% (v/v) glycerol]. The mixture was centrifuged at 14,000 × *g* at 4˚C for 15 min. The supernatant containing nuclear proteins was collected and stored at −80˚C for western blot analysis.

For enzyme linked immunosorbent assay (ELISA), whole blood was collected and placed at 4˚C overnight. The supernatants were collected after centrifugation at 1000 × *g* and 4˚C for 15 min, and stored at −80˚C for further analysis.

### 2.5 ELISA

ELISA was performed according to previous studies (Chen et al., 2020c; He et al., 2020). The supernatant was used to evaluate the TNF‐α, IL‐1β, and IL‐6 protein levels using available kits according to the manufacturer's protocol (cat. nos. EK0393, EK0412, and EK0526; Wuhan Boster Biological Technology, Ltd.). The reaction product was quantified using a microplate reader (BioTek Instruments, Inc.). The optical density (OD) value was detected at the wavelength of 450 nm.

### 2.6 Western blot analysis

Western blot analysis was performed according to previous studies (Chen et al., 2015; Chen et al., 2020a; Chen et al., 2020b; Jiang et al., 2016; Li et al., 2017; Tan et al., 2021; Yi et al., 2021). The tissue lysates mixed with a sample loading buffer was heated at 95˚C for 15 min. Protein samples were subjected to 10% SDS‐PAGE and electroblotted onto polyvinylidene difluoride membranes (EMD Millipore). After being incubated in 5% bovine serum albumin diluted in Tris‐HCl buffer saline supplemented with 0.1% Tween‐20 (TBST, pH 7.4) for 1 h to block nonspecific protein binding sites, the membranes were incubated overnight at 4˚C with one of the following antibodies: Rabbit anti‐TH antibody (1:1,000; ab137869; Abcam), rabbit anti‐NF‐κB p65 antibody (1:1,000; ab16502; Abcam), rabbit anti‐PCNA antibody (1:1,000; ab18197; Abcam) and rabbit anti‐β‐actin antibody (1:2,000; ab8227; Abcam). After washing the membrane with 0.1% TBST 3 times for 5 min each at RT, horseradish peroxidase‐conjugated goat anti‐rabbit secondary antibodies (1:10,000; ab97051; Abcam) diluted in TBST were incubated at RT for 1.5 h. Next, the membranes were washed in 0.1% TBST three times for 5 min each at RT. The immunoreactive bands were visualized by an enhanced chemiluminescence kit (cat. no. 170–5061; Bio‐Rad Laboratories, Inc.). The signal intensities were quantified by ImageJ 5.0 software.

### 2.7 Statistical analysis

All statistical analyses were performed using GraphPad Prism 6 software (GraphPad Software, Inc.). Data were expressed as the mean ± SD and analyzed with one‐way ANOVA followed by a post hoc Bonferroni test. *p *< 0.05 was considered to indicate a statistically significant difference.

## 3 RESULTS

### 3.1 Dietary Trp in the diet ameliorates impaired motor function in rotenone‐treated rats

To investigate the protective effects of dietary Trp on motor function in rotenone‐treated rats, an open field test was performed, and the total length and average speed were calculated.

**FIGURE 1:**
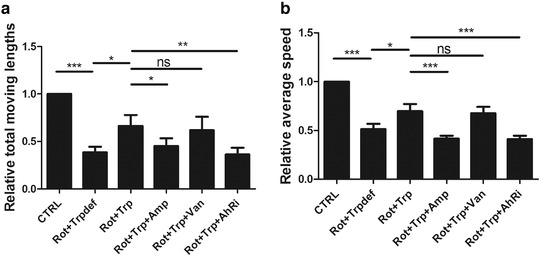
The effect of dietary tryptophan (Trp) on motor recovery in rotenone‐treated rats was investigated by open field test. Dietary Trp improves motor deficits indicated by (a) total moving lengths and (b) average speed in rotenone‐induced Parkinson's disease (PD) rats (***p < .001, **p < .01, *p < .05, *n* = 6/subgroup). NS, no significance

As shown in Figure 1, in comparison with the CTRL group, Rot+Trpdef rats exhibited motor dysfunction, including shorter lengths and lower average speed. However, Rot+Trp rats displayed significantly improved performance compared with that of Rot+Trpdef rats (*p* = .039, *p* = .011). After using ampicillin, motor dysfunction was observed in the Rot+Trp+Amp group, but after using vancomycin, motor dysfunction was significantly improved in the Rot+Trp+Van group. Moreover, after using CH223191, an inhibitor of AhR, motor dysfunction was observed.

### 3.2 Dietary Trp in the diet decreases TH expression in the SN of rotenone‐treated rats

To investigate the protective effects of dietary Trp on the degeneration of dopaminergic neurons in rotenone‐treated rats, western blot analysis was performed, and the expression of tyrosine hydroxylase (TH) in the SN of rotenone‐treated rats was evaluated.

**FIGURE 2:**
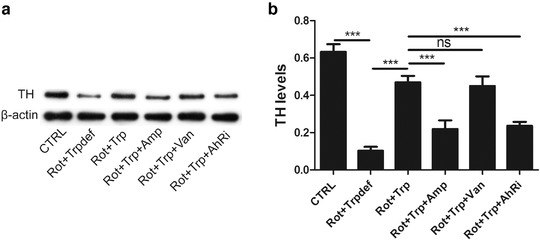
The effect of dietary tryptophan (Trp) on tyrosine hydroxylase (TH) expression levels in the SN of rotenone‐treated rats was investigated by western blot analysis. (a) Representative western blot images of TH. (b) TH was upregulated in response to treatment with dietary Trp (***p < .001, *n* = 6/subgroup). NS, no significance

As shown in Figure 2, in comparison with the CTRL group, the TH protein level was decreased in the Rot+Trpdef group. After treatment with Trp in the diet, the TH protein level was increased. After using ampicillin, the TH protein level was decreased in the Rot+Trp+Amp group. By contrast, after using vancomycin, the TH protein level was not significantly changed in the Rot+Trp+Van group. Moreover, after using CH223191, an inhibitor of AhR, the TH protein level was decreased.

### 3.3 Dietary Trp in the diet decreases the nuclear translocation of NF‐κB in the SN of rotenone‐treated rats

To evaluate the effect of dietary Trp on inflammation in the SN, western blot was performed, and the translocation of the p65 subunit of NF‐κB from the cytoplasm into the nucleus was calculated.

**FIGURE 3:**
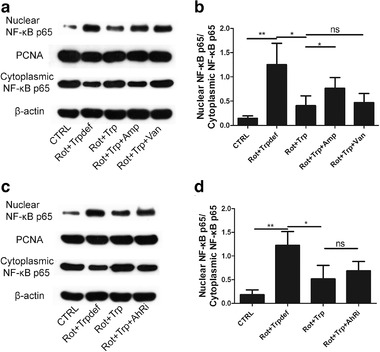
The effect of dietary tryptophan (Trp) on the nuclear translocation of NF‐κB in rotenone‐treated rats was investigated by open field test. Trp in the diet (a‐b) inhibits the nuclear translocation of NF‐κB (as indicated by the ratio of nuclear NF‐κB p65 protein levels to cytoplasmic NF‐κB p65 protein levels), but (c‐d) cannot inhibit the nuclear translocation of NF‐κB after inhibiting AhR, in rotenone‐induced Parkinson's disease (PD) rats (**p < .01, *p < .05, *n* = 6/subgroup). NS, no significance

As shown in Figure 3, in comparison with the CTRL group, the nuclear translocation of NF‐κB (indicated by the ratio of nuclear NF‐κB p65 protein levels to cytoplasmic NF‐κB p65 protein levels) was increased in the Rot+Trpdef group. After treatment with dietary Trp, the nuclear translocation of NF‐κB was decreased. After using ampicillin, the nuclear translocation of NF‐κB was increased in the Rot+Trp+Amp group, whereas, after using vancomycin, the nuclear translocation of NF‐κB was not significantly changed in the Rot+Trp+Van group. Moreover, after using CH223191, an inhibitor of AhR, the nuclear translocation of NF‐κB was increased.

### 3.4 Dietary Trp decreases inflammation in the serum of rotenone‐treated rats

To evaluate the effect of dietary Trp on inflammation in PD, the ELISA was performed, and TNF‐α, IL‐1β, and IL‐6 levels in the serum were evaluated.

**FIGURE 4:**
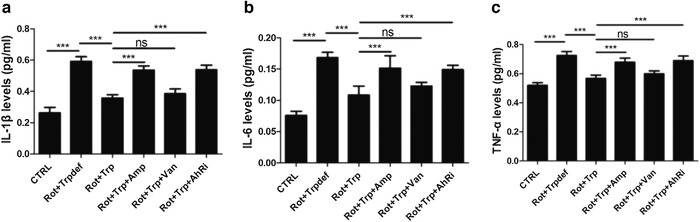
The effect of dietary tryptophan (Trp) on inflammation in the serum of rotenone‐treated rats was investigated by enzyme linked immunosorbent assay (ELISA). Trp in the diet inhibited the expression levels of proinflammatory cytokines, including (a) TNF‐α, (b) IL‐1β, and (c) IL‐6, in rotenone‐induced Parkinson's disease (PD) rats (***p < .001, *n* = 6/subgroup). NS, no significance

As shown in Figure 4, in comparison with the CTRL group, the IL‐1β, IL‐6, and TNF‐αprotein levels were increased in the Rot+Trpdef group. After treatment with dietary Trp, the IL‐1β protein levels were decreased. After using ampicillin, the IL‐1β protein levels were increased in the Rot+Trp+Amp group, while, after using vancomycin, the IL‐1β protein levels were not significantly changed in the Rot+Trp+Van group. Moreover, after using CH223191, an inhibitor of AhR, the IL‐1β protein levels were increased.

## 4 DISCUSSION

In the present study, we revealed that Trp in the diet can ameliorate impaired motor function under rotenone‐induced neurotoxicity via activating the AhR signaling pathway.

A previous study found that chronic exposure to rotenone was a risk factor for the development of PD (Betarbet et al., 2000). Administration of rotenone in rats can reproduce multiple PD‐like behavioral characters, including rigidity and hypokinesia (Wang et al., 2017). In addition, increasing evidence suggests that using rotenone to construct a PD experimental model can mimic the behavioral and neuropathological conditions of PD through selecting the degeneration of dopaminergic neurons, thus offering more advantages than other models (Johnson & Bobrovskaya, 2015). In our previous study, rotenone was used to construct an in vitro cell model to mimic the condition of PD (He et al., 2020). In the present study, a rat model was successfully constructed using rotenone, and the influence of dietary Trp on the motor deficits of PD was evaluated.

In patients with PD, motor dysfunction commonly occurs. Neurological function assessment is commonly performed to evaluate the therapeutic effect of different strategies. In the present study, we observed that Trp in the diet can ameliorate motor deficits in the rotenone‐induced rat PD model.

PD is a slowly progressive neurodegenerative disease that is associated with the degeneration of dopaminergic neurons (Vogt Weisenhorn et al., 2016). This loss of dopamine (DA) accounts for many of the symptoms that accompany the disease, including motor dysfunction, mood alterations, and cognitive impairment (Olanow et al., 2003). In the present study, we observed that Trp in the diet can upregulate TH expression in the rotenone‐induced rat PD model.

Abnormal neuronal inflammation can promote the loss of dopaminergic neurons (Yang et al., 2019). NF‐κB p65, an oxidative stress‐responsive transcription factor, modulates inflammation in multiple experimental models (Helenius et al., 1996). Under normal condition, NF‐κB p65 is segregated in the cytoplasm (Karin & Delhase, 2000), but NF‐κB p65 translocated into the nucleus to activate the downstream proinflammatory cytokines (Oeckinghaus & Ghosh, 2009). In the present study, we found that Trp in the diet can decrease the translocation of NF‐κB in rats induced by rotenone via the AhR pathway.

Proinflammatory cytokines, including TNF‐α, IL‐1β, and IL‐6, are found in either cerebrospinal fluid (CSF) or in affected brain regions in PD (Nagatsu et al., 2000). Rotenone‐injected animals displayed the occurrence of neuroinflammation (Javed et al., 2020). Previous studies have indicated that the survival of dopaminergic neurons could be protected via inhibiting neuroinflammatory responses (Furuyashiki, 2012). In the present study, we observed that Trp in the diet can inhibit the expression of NF‐κB, IL‐1β, IL‐6, and TNF‐α in rotenone‐induced rats via the AhR pathway.

## 5 CONCLUSION

Taken together, these results may suggest that Trp in the diet modulates the AhR pathway to ameliorate impaired motor function in PD, laying the foundation for Trp to be a novel candidate for the treatment of PD.

Although the results in the current study look promising, this study still exhibited some limitations. More sophisticated approaches are no doubt needed to be conducted on the female animals to see whether Trp in the diet can also ameliorate motor deficits effectively.

### PEER REVIEW

The peer review history for this article is available at https://publons.com/publon/10.1002/brb3.2226.

## AUTHOR CONTRIBUTIONS

Zijian Xiao and Heng Wu conceived and designed the experiments. Yilin Wang, Shuangxi Chen, Jian Tan, Yijiang Gao, Hongye Yan, Yao Liu, and Shanqing Yi performed the experiments and analyzed the data. Yilin Wang contributed to reagents/materials/analysis tools. Zijian Xiao, Heng Wu, and Shuangxi Chen wrote the paper.

## CONFLICT OF INTEREST

The authors declare that there is no conflict of interest.

## Data Availability

The data that support the findings of this study are available from the corresponding author upon reasonable request.
